# Recent Progress of Layered Perovskite Solar Cells Incorporating Aromatic Spacers

**DOI:** 10.1007/s40820-023-01141-2

**Published:** 2023-07-05

**Authors:** Yuping Gao, Xiyue Dong, Yongsheng Liu

**Affiliations:** 1 The Centre of Nanoscale Science and Technology and Key Laboratory of Functional Polymer Materials, Institute of Polymer Chemistry, College of Chemistry, Nankai University, Tianjin, 300071 People’s Republic of China; 2 Renewable Energy Conversion and Storage Center (RECAST), Nankai University, Tianjin, 300071 People’s Republic of China

**Keywords:** Layered perovskite solar cells, Aromatic spacers, Quantum and dielectric confinement effects, Charge transport, Efficiency and stability

## Abstract

Layered two dimensional (2D) or quasi-2D perovskites are emerging photovoltaic materials due to their superior environment and structure stability in comparison with their 3D counterparts. The typical 2D perovskites can be obtained by cutting 3D perovskites along  < 100 >  orientation by incorporation of bulky organic spacers, which play a key role in the performance of 2D perovskite solar cells (PSCs). Compared with aliphatic spacers, aromatic spacers with high dielectric constant have the potential to decrease the dielectric and quantum confinement effect of 2D perovskites, promote efficient charge transport and reduce the exciton binding energy, all of which are beneficial for the photovoltaic performance of 2D PSCs. In this review, we aim to provide useful guidelines for the design of aromatic spacers for 2D perovskites. We systematically reviewed the recent progress of aromatic spacers used in 2D PSCs. Finally, we propose the possible design strategies for aromatic spacers that may lead to more efficient and stable 2D PSCs.

## Highlights

- Layered two-dimensional (2D) perovskites are emerging photovoltaic materials with superior structural and environmental stability.
- Aromatic spacers offer unique advantages over aliphatic spacers, including higher dielectric constants, better charge transport properties, and the ability to regulate crystal arrangement, making them indispensable for constructing efficient and stable 2D perovskites.
- This review mainly focus on recent progress and achievements in developing aromatic spacer-based 2D perovskite solar cells.

## Graphical abstract

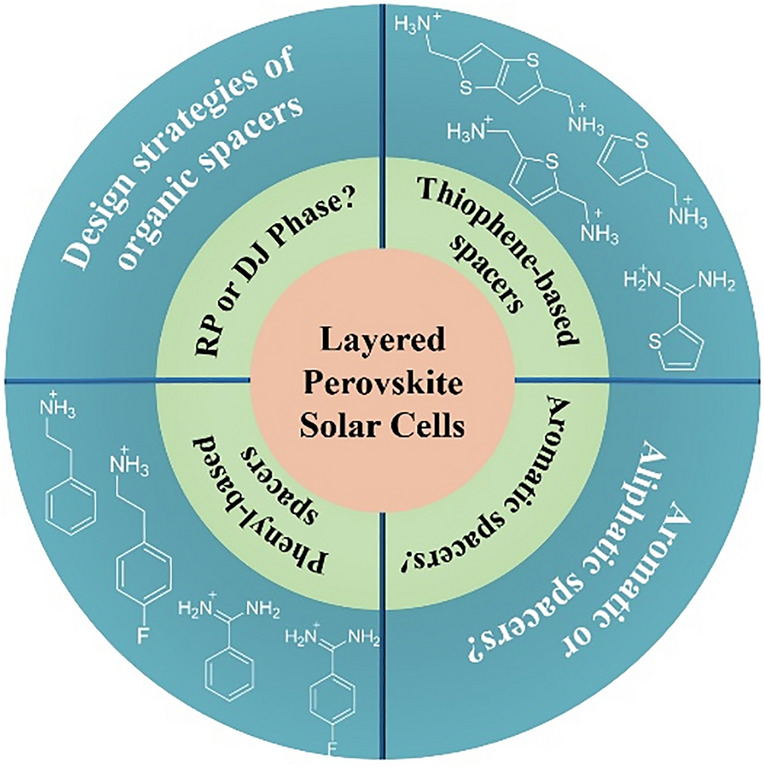

## Introduction

Organic–inorganic hybrid perovskites are considered as the emerging semiconducting materials which bring a true revolution in new-generation photovoltaic technology [1]. As shown in Fig. 2a (later), the typical chemical formula of metal halide perovskites can be represented by ABX_3_ [2], where A is a monovalent cation (CH_3_NH_3_^+^ (MA^+^), CH_2_NH = CH^+^(FA^+^) or Cs^+^); B is a bivalent metal cation (Pb^2+^or Sn^2+^), and X represents a halide anion (Cl^−^, Br^−^, I^−^). The anion parts of corner-sharing [BX_6_]^4−^ octahedra composed of B and X, in which A is located within each eight [BX_6_]^4−^ octahedra. Thanks to their dipolar properties, metal halide perovskites exhibit exceptional photophysical properties [3–5], such as high optical absorption coefficient, high carrier mobility, long carrier lifetime, and high defect tolerance. Metal halide perovskite materials have been extensively studied for applications in solar cells [1, 6, 7], light-emitting diodes [8, 9], field-effect transistors [10], photodetectors [11], and so on.

Perovskites were first utilized in solar cell as light sensitizers by Miyasaka and co-workers in 2009 and achieved a power conversion efficiency (PCE) of 3.8% [12]. In 2012, Park et al. achieved a PCE of 9.7% by incorporating spiro-OMeTAD hole transport material in PSCs [13]. Since then, the PCE of single junction perovskite solar cells (PSCs) has experienced a rapid progress and reached a certified value of 25.8% [14], which is comparable to that of commercialized silicon solar cells. However, the inferior stability under heat, moisture, light and electric field is the fatal drawback hampering the industrialization process of this technology [2, 15, 16]. Two-dimensional (2D) (including quasi-2D perovskites) recently attracted much attention due to their excellent environment and structure stability [2, 17, 18]. The typical 2D perovskites are usually visualized to cut 3D perovskites along  < 100 >  orientation by introducing bulky organic spacers [19, 20], such as n-butyl ammonium (BA) [17, 21–24], phenethlyammonium (PEA) [25] and 2-thiopenemethlyammonium (ThMA) [26]. The bulky organic spacers in 2D perovskites provide a barrier to prevent surface water adsorption and improve the environmental stability of perovskites [2].

**Fig. 1:**
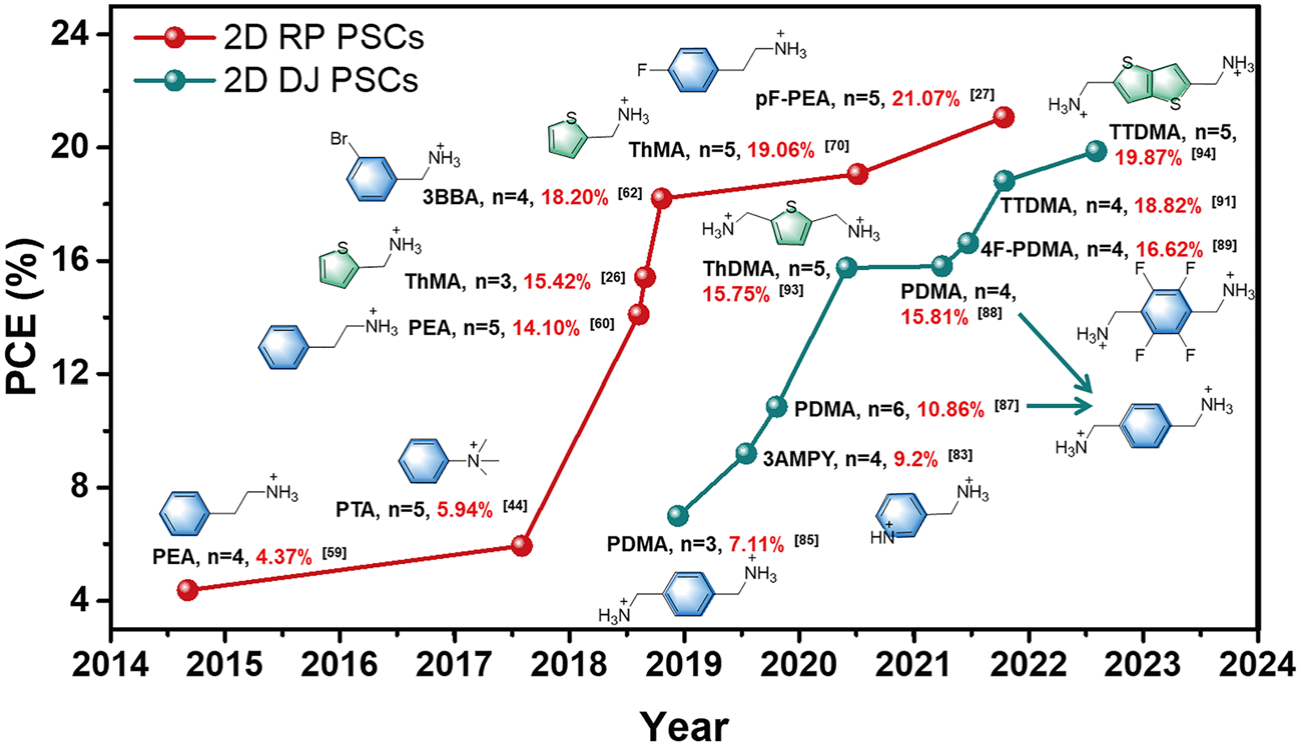
PCE evolution of Ruddlesden-Popper (RP) and Dion-Jacobson (DJ) PSCs (average n ≤ 6) based on aromatic organic spaces

Although the environmental stability of perovskites has been dramatically improved by constructing 2D perovskites, the PCE of 2D PSCs is still shown inferior performance. Figure 1 shows the evolution of record PCEs for both Ruddlesden-Popper (RP) and Dion-Jacobson (DJ) 2D PSCs based on the aromatic spacers. The corresponding photovoltaic parameters are summarized in Table 1. So far, the highest PCEs of 2D RP [27] and DJ [28] PSCs have risen all the way up to 21% and 19%, respectively. However, they are still much lower than that of state-of-the-art 3D PSCs. The inferior device performance of 2D PSCs could be ascribed to the formation of nature multiple quantum well (QW) structures due to the incorporation of insulated organic spacers [29–31]. In general, the dielectric constant of the organic layer is much lower than that of the inorganic layer leading to the strong quantum and dielectric confinement effect, which increase the exciton binding energy (*E*_b_) of 2D perovskites [32]. Moreover, the photogenerated carriers are primarily confined in the QW. The characteristic of QW that carriers can only transfer at one single “well” layer hinders efficient charge transport from one “well” to another in 2D perovskites. Note that both the QW structure and the dielectric confinement effect of 2D perovskite can be adjusted by tuning organic spacers with different energy levels, which will affect the crystal structure (inorganic octahedral distortion, interlayer distance, etc.), optoelectronic properties (band gap, exciton binding energy, charge mobility etc.), and film quality (grain size, crystal orientation, stability, etc.) of 2D perovskite [2]. Hence, the reasonable selection and innovation of organic spacers plays a crucial role to improve the efficiency of 2D PSCs.

**Table 1:** The record efficiency of 2D PSCs (n ≤ 6) based on aromatic spacers and their corresponding photovoltaic parameters

Aromatic spacer	2D Perovskite	Device structure	V_oc_ (V)	J_sc_ (mA cm^−2^)	FF (%)	PCE (%)	References
PEA	(PEA)_2_(MA)_2_Pb_3_I_10_	FTO/c-TiO_2_/perovskite/spiro-OMeTAD/Au	1.18	6.72	60	4.73	[33]
PTA	(PTA)_2_(MA)_4_Pb_5_I_16_	FTO/c-TiO_2_/perovskite/spiro-OMeTAD/Au	0.82	10.46	69	5.94	[34]
PEA	(PEA)_2_(MA)_4_Pb_5_I_16_	ITO/PEDOT:PSS/perovskite/PCBM/BCP/Ag	1.19	15.8	75	14.1	[35]
ThMA	(ThMA)_2_(MA)_2_Pb_3_I_10_	ITO/PEDOT:PSS/perovskite/PCBM/BCP/Ag	1.07	18.89	76	15.42	[26]
3BBA	(3BBA)_2_(MA)_2_Pb_3_I_10_	ITO/PTAA/perovskite/PCBM/Cr/Au	1.23	18.22	81	18.2	[36]
ThMA	(ThMA)_2_(FA)_4_Pb_5_I_16_	ITO/PEDOT:PSS/perovskite/PCBM/BCP/Ag	1.07	23.39	76	19.06	[37]
pF-PEA	(pF-PEA)_2_(FA_0.3_MA_0.7_)_4_Pb_5_I_16_	ITO/PTAA/perovskite/PCBM/BCP/Au	1.18	22.45	79	21.07	[27]
PDMA	(PDMA)FA_2_Pb_3_I_10_	FTO/c-TiO_2_/mp-TiO2/perovskite/spiro-OMeTAD/Au	0.84	11.49	72	7.11	[38]
3AMPY	(3AMPY)(MA)_3_Pb_4_I_13_	ITO/PEDOT:PSS/perovskite/C60/BCP/Ag	1.08	14.34	59	9.2	[39]
PDMA	(PDMA)MA_5_Pb_6_I_19_	ITO/PEDOT:PSS/perovskite/PCBM/Bphen/Ag	1.08	20.9	48	10.86	[40]
PDMA	(PDMA)(MA)_3_Pb_4_I_13_	FTO/c-TiO_2_/perovskite/spiro-OMeTAD/Au	1.15	21.1	62	15.81	[41]
4F-PDMA	(4F-PDMA)(MA)_3_Pb_4_I_13_	ITO/PEDOT:PSS/perovskite/PCBM/BCP/Ag	1.1	19.58	77	16.62	[42]
TTDMA	(TTDMA)MA_3_Pb_4_I_13_	ITO/PEDOT:PSS/perovskite/PCBM/BCP/Ag	1.03	22.38	81	18.82	[43]
TTDMA	(TTDMA)MA_4_Pb_5_I_16_	ITO/PEDOT:PSS/perovskite/PCBM/BCP/Ag	1.08	21.35	86	19.87	[44]

**Fig. 2:**
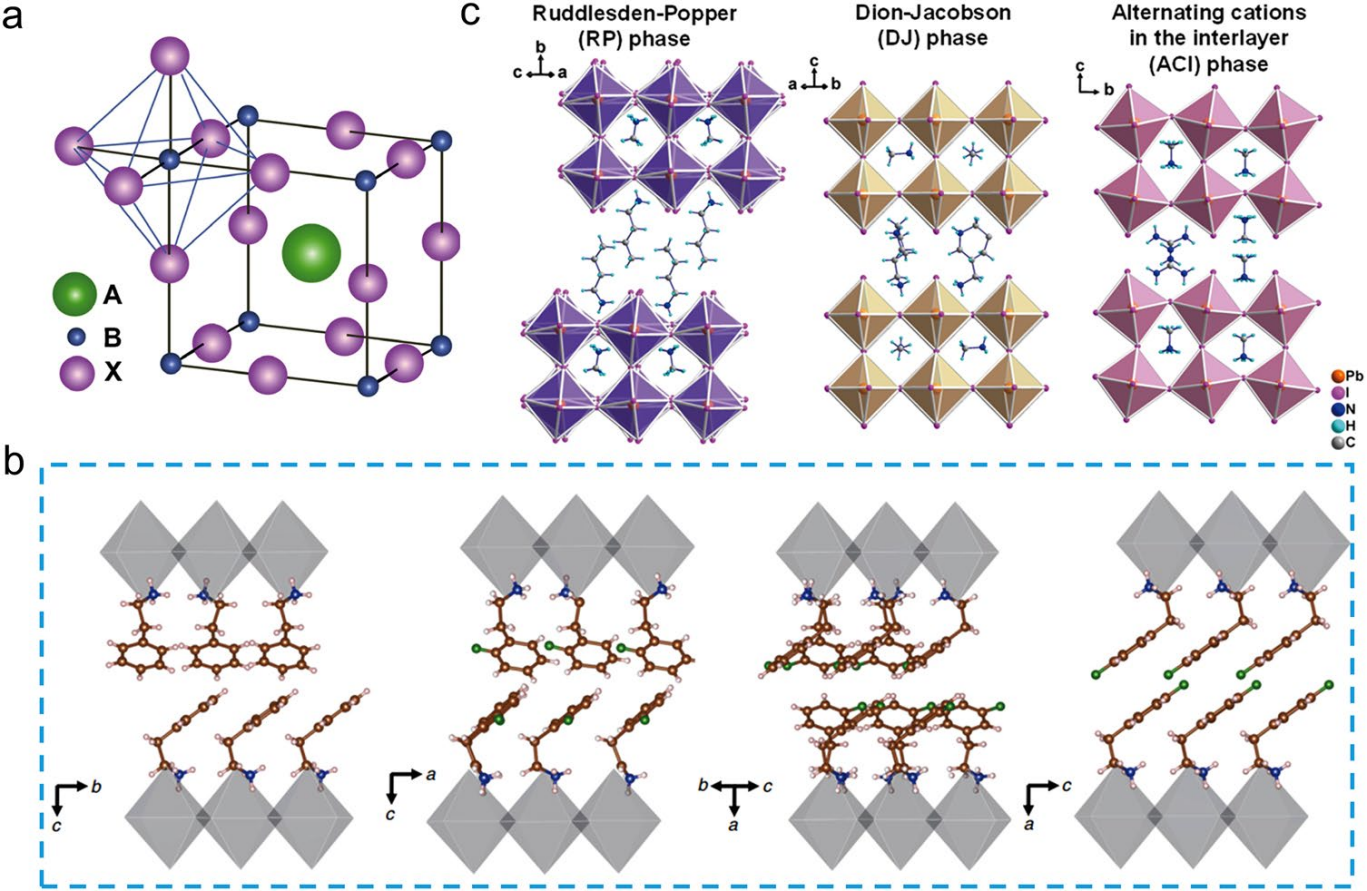
**a** ABX_3_ perovskite structure [59]. **b** Crystal structures of 2D perovskite showing the different packing arrangements induced by different PEA-based spacers [56]. **c** The crystal structures of Ruddlesden-Popper (RP), Dion-Jacobson (DJ) and alternating cation interlayer (ACI) 2D perovskite [2]

*Aromatic spacers or Aliphatic spacers?* The organic spacers used in 2D PSCs can be typically categorized as aliphatic spacers [17, 21, 45, 46] and aromatic spacers [47, 48], which are fundamentally different in their chemical and physical properties. Aliphatic spacers can be further divided into linear [21, 49, 50], branched, or cyclic [51–53] configurations. Linear aliphatic spacers like BA [21] cations are commonly used in 2D perovskite due to their excellent solubility and low steric hindrance for perovskite film processing. However, aliphatic spacers have insulating properties that increase quantum and dielectric confinement effects. On the other hand, aromatic spacers are believed to benefit charge transport in 2D perovskite films. Aromatic spacers have the following unique advantages over aliphatic spacers [34]: (1) *Higher dielectric constants*. Aromatic spacers with high dielectric constants can reduce the dielectric mismatch between the organic and inorganic layers, which improves charge transport and PCE of 2D PSCs [2, 20, 54]. (2) *Bulky conjugated structure*. The bulky conjugated structure of aromatic spacers confers enhanced stability of 2D perovskites [34, 55] and regulate arrangement and orientation of perovskite crystals through the π-π interactions as well as hydrogen bonding (Fig. 2b) [56, 57]. Aliphatic spacers with soft organic tails provide greater flexibility and weaker interactions with each other during 2D perovskite film formation. In contrast, the aromatic spacers with conjugated structures results in more intensive interaction via π-π interactions and hydrogen bonds, restraining the slip of the inorganic slabs along the van der Waals gap direction, resulting in a more stable 2D perovskite structure [57]. (3) *The red-shifted absorption spectra*. Due to the easily tunable bandgap of organic spacers, the aromatic spacers may contribute to light absorption in the visible range [34]. Given these advantages, we believe that aromatic spacers will remain in an irreplaceable position for constructing 2D perovskites [58].

We note that several previous reviews on 2D perovskite materials and related devices have been reported [2, 60–66]. This review focus mainly on the recent achievements in aromatic spacer-based 2D PSCs such as phenyl derivatives, heterocyclic compounds, polycyclic aromatic compounds. Moreover, the effects of the spacer molecular structures on the photovoltaic performance of 2D PSCs are also systematically compared. Finally, we point out a view of designing aromatic spacers for efficient 2D PSCs.

## Application of Aromatic Spacers in 2D PSCs

### Aromatic Spacers for 2D RP PSCs

**Fig. 3:**
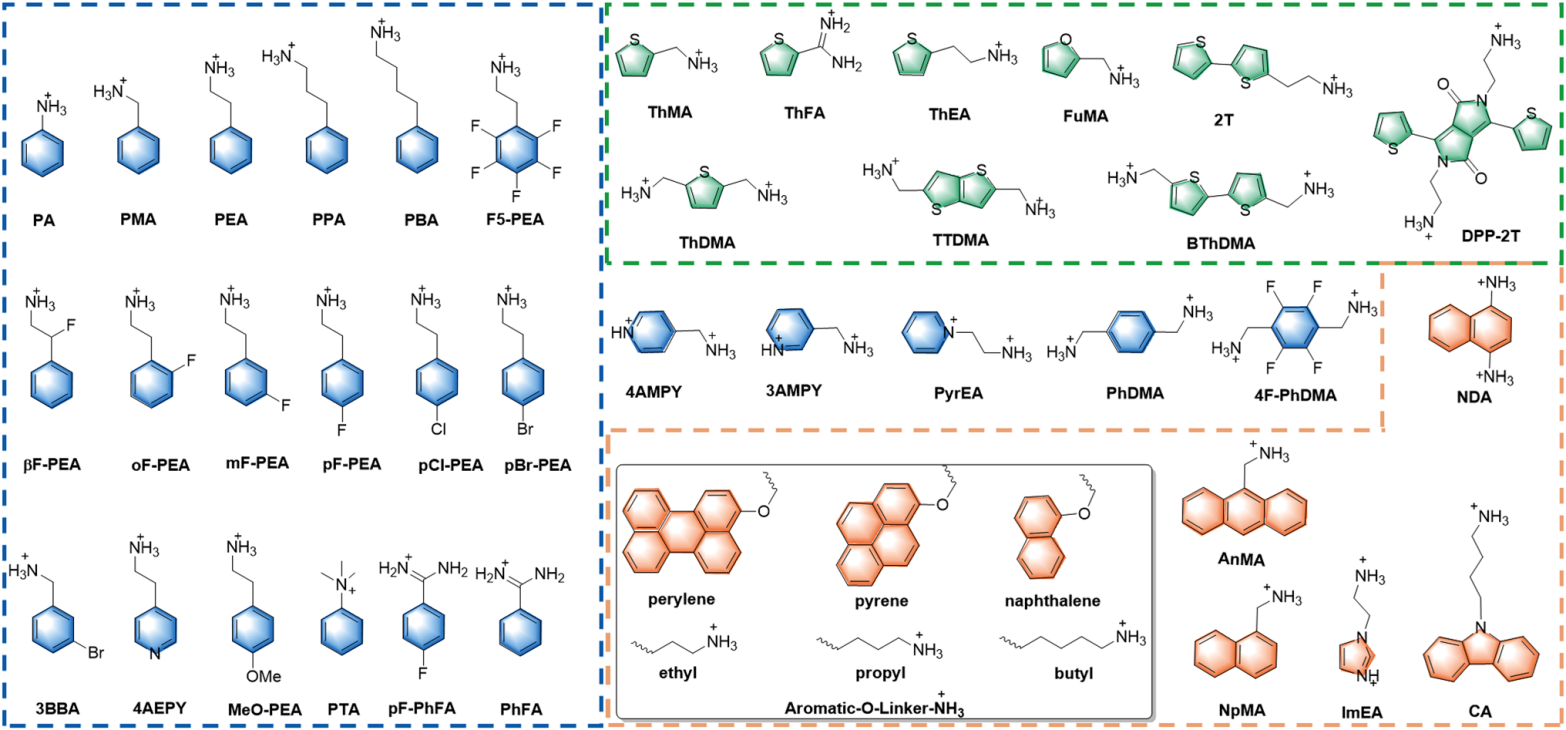
Representative aromatic organic spacers

2D PSCs can be divided into three categories, including RP, DJ and ACI types based on the number of the amino group of organic spacers (Fig. 2c). The organic spacer of 2D ACI perovskites only consists of guanidinium [28, 67, 68], we focus mainly on RP and DJ type 2D perovskites in this review. The chemical formula of RP and DJ type 2D perovskites is (L_1_)_2_A_n−1_B_n_X_3n+1_ and (L_2_)(A)_n-1_B_n_X_3n+1_, respectively [33, 51, 63, 69]. Here L_1_ is a bulky monovalent organic cation; L_2_ is a divalent organic cation; A, B, and X are the same compounds as the 3D perovskites mentioned earlier. Monovalent organic spacers are usually used to construct 2D RP perovskite films where the amine group of spacers interacts with the inorganic layer. The tail of the spacer binds with each other via van der Waals interactions, forming van der Waals gaps. It is worth noting that the existence of van der Waals gaps may reduce the structural stability of 2D RP perovskite films due to weaker non-covalent interactions between the tails of aromatic spacers. Moreover, the tail group of organic spacers could directly influence their self-assembled structures which could affect the crystal orientation and phase distribution of 2D perovskite. Thus, the chemical structure of aromatic spacers plays a critical role in determining the optoelectronic properties of 2D RP PSCs. The representative monoammonium aromatic spacers used in 2D RP PSCs are shown in Fig. 3.

#### Phenyl-Based Spacers

**Fig. 4:**
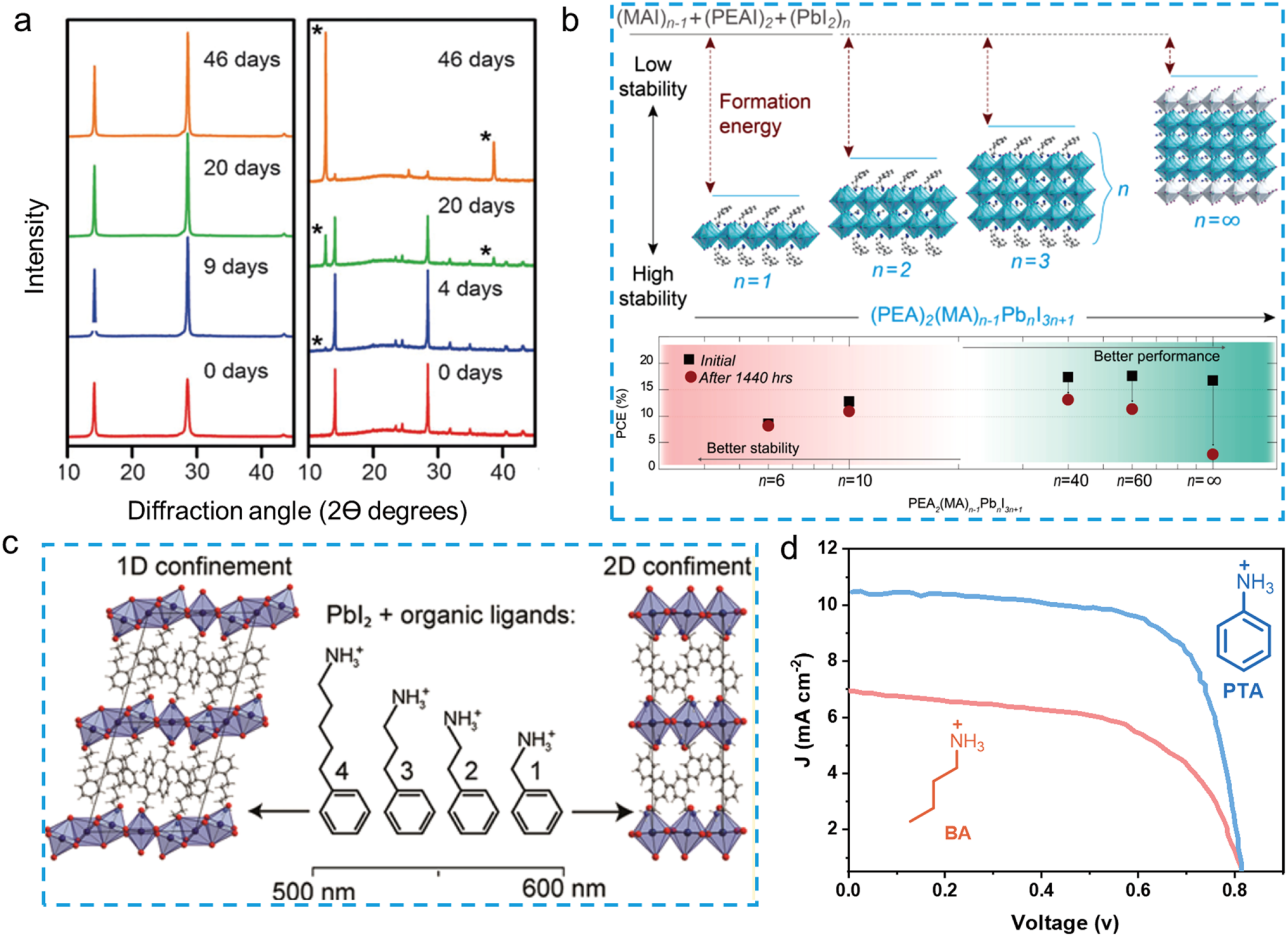
**a** XRD patterns of films of (PEA)_2_(MA)_2_Pb_3_I_10_ (left) and MAPbI_3_ (right) which were exposed to 52% relative humidity [35]. **b** Energetics of perovskite formation and stability; Device performance as a function of n value, which shows that increased performance was achieved with increased n value; however, in the meantime, stability was decreased [25]. **c** Chemical structure of phenylalkylammonium spacer and their corresponding crystal structure of low dimensional peroskites [71]. **d** *J*-*V* curves of champion devices prepared with PAI and BAI [54]

Phenylethylammonium (PEA) is the first reported aromatic spacer for 2D PSCs. In 2014, the 2D RP PSC with the structure of (PEA)_2_(MA)_2_Pb_3_I_10_ was first reported by Karunadasa and coauthors [35], and achieved a PCE of 4.73% with an open-circuit voltage (*V*_*OC*_) of 1.18 V. Interestingly, this 2D (PEA)_2_(MA)_2_Pb_3_I_10_ perovskite film could be fabricated under ambient conditions due to its superior humidity resistance. When the film was exposed to 52% relative humidity, the diffraction peaks of the X-Ray diffraction (XRD) showed no apparent change after 42 days, whereas the 3D perovskite (MAPbI_3_) was completely decomposed into the PbI_2_, suggesting the superior moisture stability of 2D (PEA)_2_(MA)_2_Pb_3_I_10_ perovskite (Fig. 4a). To explore the origin of the stability of 2D perovskites, Sargent et al*.* reported the formation energy of PEA_2_(MA)_n‑1_Pb_n_I_3n+1_ perovskite films with different n values (n = 1–3) [25]. The results showed that the 2D perovskites exhibit higher formation energy compared to their 3D counterpart, which explained the improved stability (Fig. 4b). Notably, the van der Waals force between organic spacers and the higher energy required to remove spacers from the perovskite were also responsible for the increased stability. To increase the crystallinity of 2D perovskite films, Alex et al*.* innovatively introduced the additives of ammonium thiocyanate (NH_4_SCN) and ammonium chloride (NH_4_Cl) to the 2D perovskite precursor (n = 5). They obtained an efficient device with PCE of 14.1% benefitting from the preferred crystal orientation of 2D perovskite perpendicular to the substrate, which can promote the charge transportation and collection from the bottom to the top in photovoltaic devices [70]. Zhang et al. also reported efficient 2D PSCs with a PCE of 14.3% by directly using in situ formed PEA cation [48]. Recently, Heejoo Kim et al. have developed a simple post-treatment method to improve the efficiency and stability of PSCs based on 2D RP perovskite (PEA_2_MA_4_Pb_5_I_16_) by using zwitterionic n-tert-butyl-α-phenylnitrone (PBN) as a passivation material. The PBN enhances the vertical charge transport of the 2D perovskite by passivating surface and grain boundary defects. Moreover, the PBN passivation strongly suppresses ion migration in the MA-based RP perovskite resulting in the high efficiency of 20.05% obtained with excellent operating stability [36]. The successful application of PEA in the 2D PSCs has inspired researchers’ interest in exploring similar organic spacers. Palstra et al*.* systematically studied the effect of the phenylalkylammonium cations with different alkyl chain lengths on the 2D perovskite single crystal structures [71]. The 2D perovskite single crystals based on benzylamine (PMA), phenethylamine (PEA), 3-phenyl-1-propylamine (PPA), and 4-phenyl-1-butylamine (PBA) were obtained. They found that phenylalkylammonium spacer with longer alkyls (PPA and PBA) yielded both corner- and face-sharing of [PbI_6_]^4−^ octahedra, which formed new compounds with chemical formula of (PPA)_3_Pb_2_I_7_ and (PBA)_3_Pb_2_I_7_, respectively. Only shorter alkyls (PMA and PEA) could form the classical corner-sharing A_2_PbI_4_ structure (Fig. 4c). Based on the above findings, anilinium cation (PAI) with the shortest alkyls was used in 2D PSCs by Barea et al*.* [54]. In comparison with butylammonium iodide (BAI), the devices of PA-based 2D perovskite exhibited a higher photocurrent (*J*_*SC*_) and much improved PCE (Fig. 4d), which could be attributed to the decreased dielectric confinement effect and enhanced charge transport abilities benefitting from the higher dielectric constant of aromatic organic spacer.

**Fig. 5:**
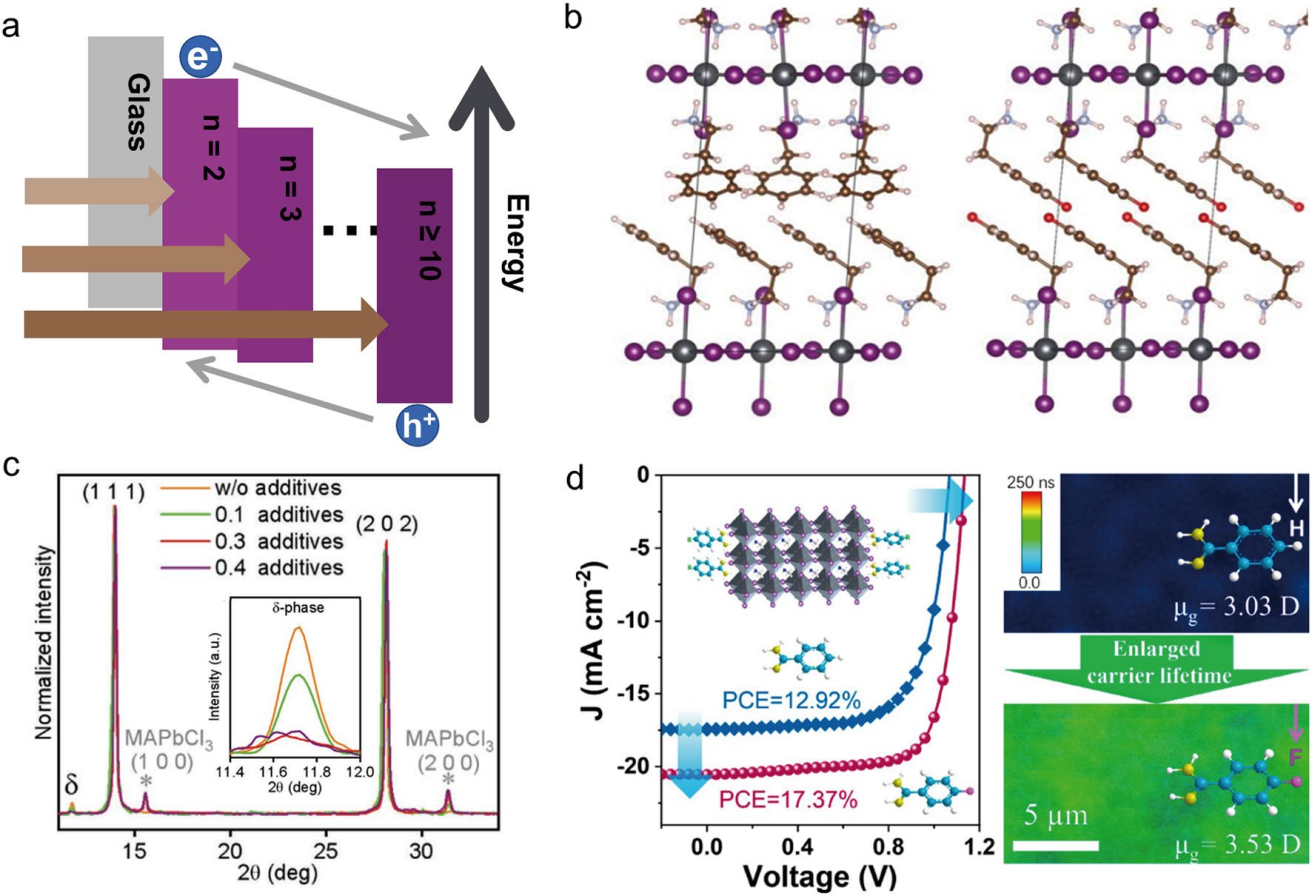
**a** Energy level alignment of 2D RP perovskite film with different n values [74]. **b** (PEA)_2_PbI_4_-and (pF-PEA)_2_PbI_4_-based single-crystal structure [75]. **c** XRD patterns of pure FA 2D RP perovskite films with different chloride additives concentration; inset: the enlarged diffraction peak of δ-phase of FAPbI_3_ [27]. **d** *J − V* curves of PhFA-Pb and pF-PhFA-Pb devices; time-resolved confocal fluorescence microscopy (TCFM) images of the PhFA-Pb and pF-PhFA-Pb films [37]

Except for the commonly used organic spacers of PMA and PEA in 2D perovskite, their derivatives are also emerging constantly. The modified PEA by introducing the halogen atoms to the phenyl ring positively affects the perovskite film quality, and the humidity stability of corresponding perovskite devices. For example, 3-bromobenzylammonium iodide (3BBAI) was developed by Huang et al*.* to fabricate 2D perovskite solar cells which exhibited excellent moisture resistance [72]. After the 3BBA-based film were immersed into water for 60 s, no apparent XRD diffraction peaks decline was observed due to their superior hydrophobic nature and improved film crystallinity. Notably, the researchers found that the low-n phase components were mainly located at the bottom side along the direction perpendicular to the substrate, followed by the large-n components at the top of the film, which was caused by heteroatom in 3BBAI organic spacer. The lower Urbach energy (*E*_u_) observed in 3BBAI-based 2D perovskite film indicated that it exhibits a high degree of electronic order. As a result, an impressive PCE of 18.20% was achieved. In 2019, You et al. used a mixture of perfluorophenethylammonium (F5-PEA) and PEA (1:1 molar ratio) as organic spacers to synthesize 2D PSCs (n = 4) with high stability and performance [57]. They found that introducing the strong quadrupole–quadrupole interaction between F5-PEA and PEA could significantly improve not only their structure stability but also the device stability. Alex et al. reported stabilized 2D perovskite films based on pF-PEAI and MeO-PEAI with high moisture tolerance [73]. They found that the 2D perovskites film with larger size (MeO-PEAI) or more hydrophobic (pF-PEAI) cations exhibited excellent moisture stability. The best photovoltaic efficiency was achieved by the pF-PEA-based 2D PSCs, which showed a PCE of 14.5%, which is higher than that of the 13.2% and 9.9% for PEA- and MeO-PEA-based devices, respectively This result was ascribed to the large cation of MeO-PEA, which destroyed the organized arrangement of organic spacers, resulting in poor crystallinity. On the other hand, the fluorine in pF-PEA spacer could regulate the arrangement and orientation of the 2D perovskite. Meanwhile, Shao et al*.* also demonstrated the great potential of fluorinated organic spacers for application in 2D PSCs and systematically investigated the effect of fluorination on the performance of 2D RP PSCs [74]. They found that the 2D RP perovskite fabricated by fluorinated PEAI cation (F-PEAI) exhibited higher film quality, vertical crystal growth direction, reduced exciton binding energy, and superior environmental stability compared to the PEA-based 2D perovskite film. Note that the distinct phase distribution with the increasing n number from the bottom to the top surface was observed in the pF-PEA-based 2D perovskite films, forming a cascade energy band diagram (Fig. 5a), which facilitate charge transport. Eventually, an improved efficiency of 17.3% was achieved.

The single crystal structure of pF-PEA-based 2D perovskite was obtained by Zhu and coworkers (Fig. 5b) [75]. In comparison with PEA-based 2D crystal perovskite, the centroid distances between two pF-PEA spacers in corresponding 2D perovskite single crystal are reduced, resulting in more ordered packing and enhanced charge transport properties. The results clarified the influence of fluorination on the improved the film quality and the enhanced photovoltaic performance of corresponding 2D PSCs. The different fluorinated positions of benzene can also significantly affect the photovoltaic performance of 2D PSCs. The fluorine group at different positions of the benzene ring in the PEA spacer for 2D PSCs (n = 4) was also systematically investigated [56]. Of the series 2D perovskite crystal structures (PEA, oF-PEA, mF-PEA, and pF-PEA), the pF-PEA-based 2D perovskite exhibits the least orientational disorder of organic cations, resulting in the best device performance, followed by mF-PEA, PEA, and oF-PEA. It can be explained by the different formation energies of these 2D perovskite films. The (pF-PEA)_2_PbI_4_ exhibits the most favorable formation energy due to the intensive noncovalent interactions of the organic cations. In addition, in compared with other 2D perovskites (PEA-, pCl-PEA- and pBr-PEA-based), pF-PEA-based 2D perovskite possesses narrower optical band gaps, which exhibits the reduced *E*_b_, which could be attributed to the weaker structure distortion of the inorganic sheets [76]. Based on the research described above, Zhang et al*.* reported pure FA-based 2D PSCs, (pF-PEA)_2_(FA)_4_Pb_5_I_16_ (n = 5), containing methylammonium chloride (MACl) and lead chloride (PbCl_2_) as the additives to modify the crystal orientation and film quality, achieving a record-efficiency of 21.07% (certified at 20%) [27]. In this system, the traces MAPbCl_3_ clusters formed in the film could assist the crystallization of black phase α-FAPbI_3_ and suppress the formation of the yellow δ-phase (Fig. 5c), which is beneficial for light absorption. According to the results of grazing incidence wide-angle X-ray scattering (GIWAXS) and transient absorption (TA) spectroscopy, the black α-FAPbI_3_ phase was generated throughout the whole 2D perovskite layer. Moreover, the low-n phase 2D perovskite crystals at the bottom tend to grow perpendicular to the subtract, which could facilitate the charge transportation in 2D (pF-PEA)_2_(FA)_4_Pb_5_I_16_ PSCs. The introduction of fluorine atom into the alkyl chain of PEA can change the dipole moment of the spacer, which enhance the interactions between the organic and inorganic layers, and further improve the charge separation ability and crystal quality of the films. Based on this idea, a novel *β*-fluorine-substituted phenylethylammonium (βF-PEA) was synthesized for 2D RP perovskite [77]. Compared to the PEA (μ = 1.26 Debye), βF-PEA had a lager electric dipole moment (μ) of 1.71 Debye, which could reduce the exciton binding energy and increase the PCE of 2D PSCs to 19.11%. Notably, owing to the enhanced interaction between the βF-PEA and [PbI_6_]^4−^ octahedra, the phase stability of βF-PEA-based 2D perovskite was also dramatically improved. When exposed to humid conditions, βF-PEA-based 2D perovskite film exhibited unchanged XRD diffraction peaks, indicating the superior phase stability. However, the unfavorable α → δ phase transformation was observed for PEA-based 2D perovskite film. With the erosion of water molecules, the mixed phase spontaneously formed a more stable low-dimensional phase, which damaged the PCE of the devices.

Apart from PEA and their derivatives, many other phenyl-based spacers have been reported for the application of 2D RP PSCs, such as benzamidine (PhFA), para-fluorobenzamidine (pF-PhFA) and phenyltrimethylammonium (PTA). Liu et al*.* reported a class of formamidinium (FA)-based aromatic spacers, PhFA, and pF-PhFA, and investigated their effects on the photophysical and photovoltaic properties of 2D RP perovskites [37]. In contrast to PhFA-based 2D perovskite (n = 5), the pF-PhFA-based 2D perovskite exhibited high film quality, preferred vertical orientation, and improved charge carrier mobility, resulting in a superior efficiency of 17.37% (Fig. 5d) and enhanced stability. The increased crystallinity and orientation of pF-PhFA-based perovskite film was attributed to the incorporation of fluorine atoms, which enhanced the dipole moment and dielectric constant of the organic spacer and reinforced interaction between the inorganic slabs and organic spacer. In 2019, Chen et al*.* reported a phenyltrimethylammonium (PTA) spacer for 2D RP perovskites [78]. With the assistance of MACl, the (PTA)_2_(MA)_3_Pb_4_I_13_ 2D RP perovskite film exhibited ordered crystal orientation, good charge transport capability, and low density of defect states, resulting in efficient and stable PSCs with a PCE of 11.53%. Phenyl-based organic cations are the most used spacers in 2D perovskite, and the highest efficiency 2D PSCs is also based on the spacers containing phenyl group ((pF-PEA)_2_(FA)_4_Pb_5_I_16_ (n = 5), PCE = 21.07%). However, the application of phenyl-based spacers with different heteroatoms and alkyl chain lengths in 2D PSCs needs systematic research to further improve their PCEs.

#### Thiophene-Based Spacers

**Fig. 6:**
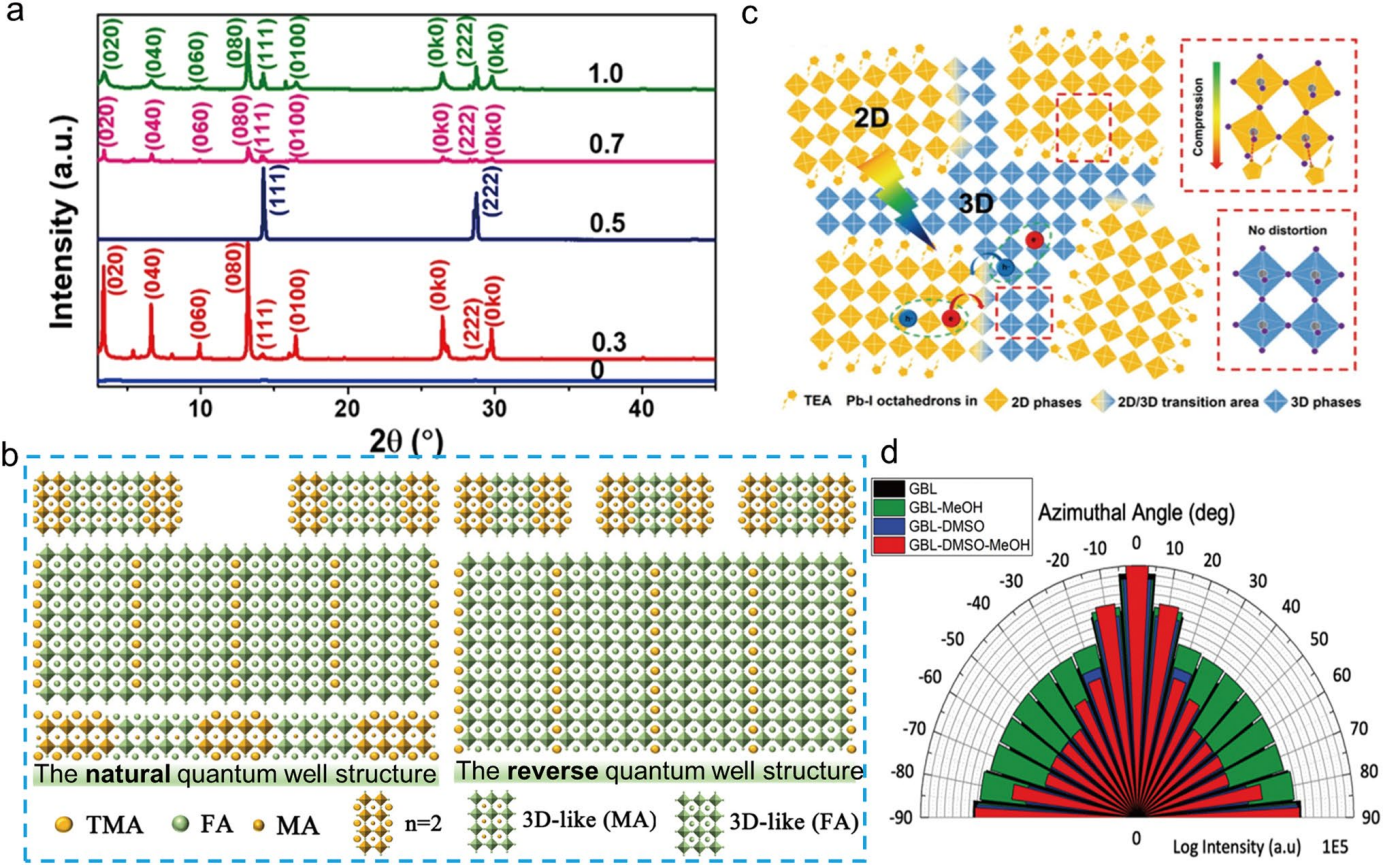
**a** XRD patterns of 2D (ThMA)_2_(MA)_n−1_Pb_n_I_3n+1_ (n = 3) films formed with different MACl/MAI weight ratios from 0 to 1 [26]. **b** Schematic diagrams of the natural quantum well structure and the reverse quantum well structure [31]. **c** Schematic illustration of 2D/3D mixed phases in TEA perovskites [81]. **d** Intensity of (111) plane corresponding to a Q_xy_ of 1.0 Å^−1^ plotted along azimuthal angle [82]

The π-conjugated thiophene unit exhibits large electron cloud density and has been widely used as constructed building blocks in organic semiconductor materials. Moreover, the S in thiophene unit could also interact with the undercoordinated Pb^2+^ through S‧‧‧Pb interactions. In 2018, Liu et al*.* first reported efficient 2D RP PSCs based on the thiophene-based organic spacer (2-thiophenemethylammonium, ThMA) [26]. A MACl assisted film-forming technique of 2D perovskite film was successfully demonstrated. With MACl as additive, the crystallinity of 2D perovskite film was dramatically improved (Fig. 6a), and achieving a dense nanorod-like films morphology, which significantly improves the charge transport capability. Eventually, the ThMA-based 2D PSCs (n = 3) achieved a record efficiency of 15.42%, and the corresponding devices exhibited improved long-term stability compared to their 3D counterparts. To further enhance 2D PSCs performance, Liu et al*.* introduced the FA component into the ThMA-based 2D perovskite to reduce the bandgap and improve the light absorption capacity [79]. The efficiencies of the (ThMA)_2_(FA)_4_Pb_5_I_16_-based 2D PSCs were improved to 16.18%. An organic salt, namely 4-(trifluoromethyl)benzylammonium iodide (tFM-PMAI), assisted crystal growth (OACG) technique was further developed to regulate the crystallization process and crystal orientation of (ThMA)_2_(FA)_4_Pb_5_I_16_ film, resulting in the suppressed non-radiative recombination loss and improved carrier mobility. Consequently, a record PCE of 19.06% was obtained. It was worth noting that the synergistic effect of IPA and tFM-PMAI was the main reason for the improved film quality and orientation of OACG-processed perovskite, which could be confirmed by the GIWAXS measurements. The (0k0) diffraction peak along the q_z_ direction was strong in the control film but almost disappeared in the OACG-processed film, indicating the vertically orientated 2D perovskite crystals. Based on the above study, Chen et al*.* used the ThMA as an organic spacer to prepare FA-MA mixed 2D perovskite films, which exhibited a quantum well reversely graded structure [31]. The quantum well reversely graded structure was defined as the low-n-value phase concentrated on the film surface and the 3D-like phase distributed inside of the film (Fig. 6b). This unique structure could weaken the quantum confinement effect resulting in the record-high efficiency of 20.12% and improved moisture stability. In addition, Yang et al*.* compared the difference between ThMA-based and the traditional phenyl spacers-based (PEA and PMA) 2D perovskite films and found that the ThMA-based 2D PSCs exhibited more excellent charge transport capability [80]. To investigate the electronic structure of 2D perovskites with ThMA, PEA and PMA spacers, the density functional theory (DFT) calculations were performed. The projected density of state (pDOS) indicated that for all ThMA-, PEA- and PMA-based 2D perovskite, the iodine and lead atoms are mainly responsible for conducting holes and electrons. However, the carbon pDOS of the ThMA spacer was closer to the VB edge than the analogue in the PEA or PMA structure, demonstrating a better hole mobility between the organic and inorganic layers which resulted in the enhanced conductivity of 2D perovskite and a better photovoltaic efficiency (PCE = 19%) for ThMA-based 2D PSCs.

Thiophene-based organic spacer, 2-thiophene ethylamine (ThEA), was also developed for 2D perovskites [81, 83]. Liang et al*.* found that the ThEA can regulate phase distribution in 2D perovskite films, promoting the spontaneous generation of 2D/3D heterojunction structures [81]. The strong Pb–S interaction could compress the perovskite lattice and facilitate the nucleation process in the perovskite precursor, which lead to a unique film morphology of the 3D-like phase distributed at the 2D perovskite grain boundary (Fig. 6c). The tiny 3D phase could provide an additional charge transport path and result in a PCE over 11%. Moreover, Qin et al*.* reported a new method by using the (ThEA)_2_(MA)_2_Pb_3_I_10_ single crystals to prepare the perovskite precursor [83]. Notably, the nonpolar cosolvent, dimethylacetamide (DMAc): toluene (TOL): hydrogen iodide (HI), was used to dissolve the single crystals and the addition of HI can reduce the interaction between PbI_2_ and DMAc, resulting in suppressed structure decomposition of the corresponding 2D single crystals. By cooperating with this method, the related 2D perovskite films exhibit a narrow phase distribution and vertical crystal orientation, resulting in a repeatable and efficient 2D PSCs. The successful application of FA as an A-site cation in 3D PSCs has drawn the researchers’ interest in developing FA-based organic spacers. Liu et al*.* first reported an FA-based spacer (2-thiopheneformamidinium, ThFA) for 2D RP PSCs [84]. By dropping the dilute isopropyl alcohol (IPA) solution containing ThFAI, MAI, and MACl onto the perovskite film surface as an anti-solvent during the spin-coating process, a precursor organic salts-assisted crystal growth (PACG) technique was developed to adjust crystal orientation, modulate the film quality and phase distribution. Using this method, the resulted 2D perovskite films shown improved film quality with enlarge grains, increased carrier mobility and suppressed non-radiative composite losses. As a result, the (ThFA)_2_(MA)_n-1_Pb_n_I_3n+1_ (n = 3) devices showed a high PCE of 16.72% for p-i-n structured 2D PSCs with negligible J-V hysteresis. The ThMA and ThFA spacers were further introduced into 2D Cs-based inorganic PSCs, achieving high-quality perovskite film with excellent phase stability [85]. It was found that introducing the thiophene-based organic spacers into inorganic perovskite films could release the inner stress of the CsPbI_3_ phase, thus significantly improving its phase stability. More importantly, the ThFA-based 2D RP perovskite, (ThFA)_2_(Cs)_n-1_Pb_n_I_3n+1_ (n = 5), exhibited more uniform phase distribution and preferred vertical crystal orientation with respect to the substrate, which resulted in a higher efficiency of 16.00% in comparison with the ThMA-based devices (PCE = 12.62%). Extending the conjugated length or adjusting the perovskite phase distribution are effective methods to reduce the quantum and dielectric confinement effect [64]. Using a bulky bi-thiophene ligand (2 T) as an organic ligand, Dou et al*.* reported a 2D PSCs based on (2 T)_2_(MA)_6_Pb_7_I_22_ with improved vertical crystal orientation (Fig. 6d), which was achieved by the mixed solvent of gamma-butyrolactone (GBL): dimethylsulfoxide (DMSO): methanol (MeOH) (v/v, 10:1:4), resulting in a PCE of 13.3% [82]. Due to the large volume of 2 T molecules, the humidity stability of the corresponding 2D device has also been significantly improved.

The highest efficiency of thiophene-based 2D PSCs reported so far is 20.12%, which is close to the phenyl-based counterpart (21.07%). Nonetheless, for thiophene group, the sulfur atom exhibited a higher polarizability and a larger electron cloud density than benzene ring [80]. This pretty much means that the thiophene-containing spacers have greater potential than phenyl-based spacers in the field of 2D PSCs. Therefore, the researchers could pay more attention to the structure-morphology-property relationship, such as the crystal structure of the thiophene-based 2D perovskites, the intermolecular interactions caused by sulfur atom in thiophene group and the photophysical behavior of the 2D perovskite film.

#### Other Polycyclic or Heterocyclic Compound-Based Spacers

**Fig. 7:**
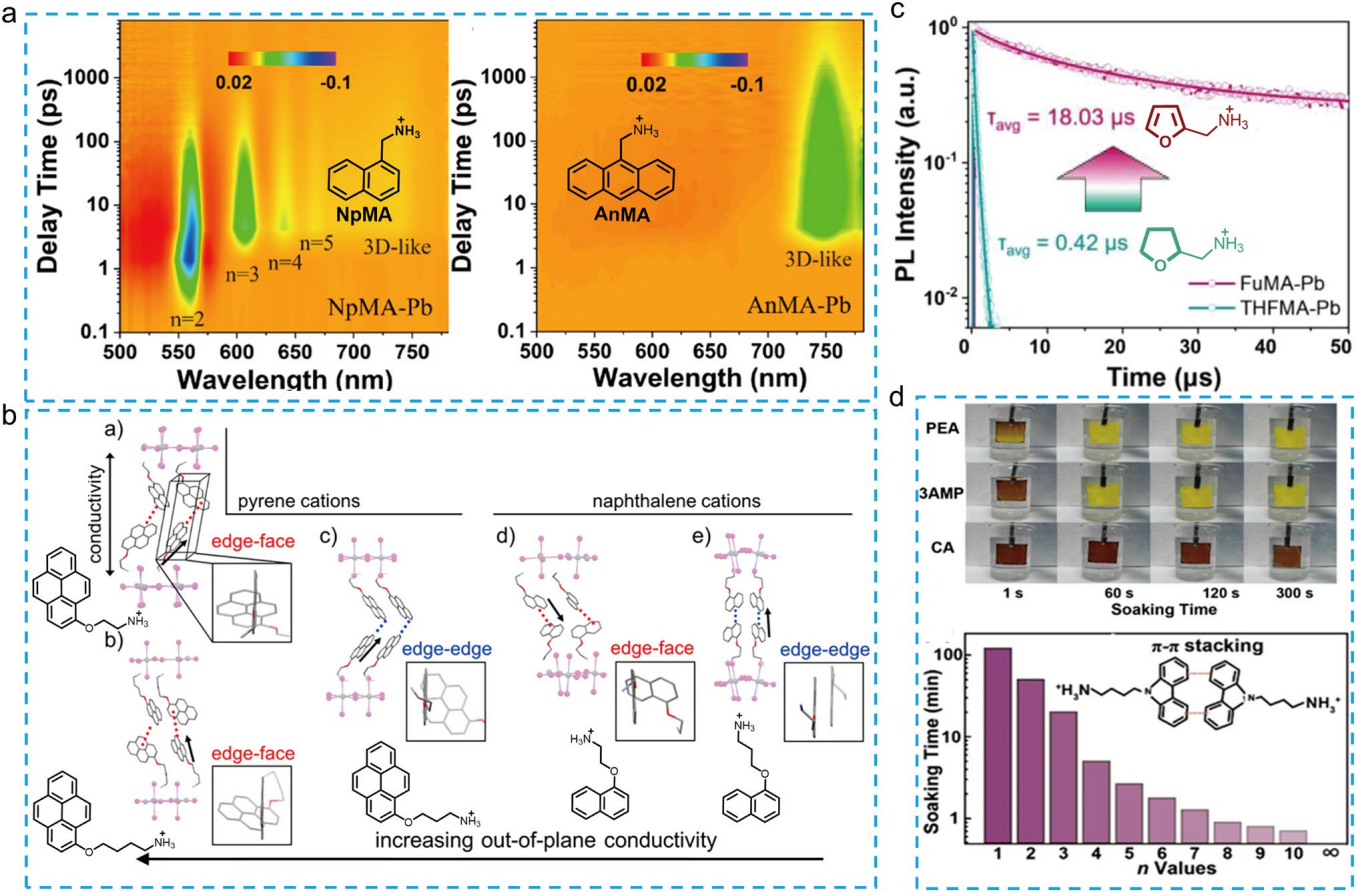
**a** Time- and wavelength-dependent TA images of the NpMA- and AnMA-based film [87]. **b** Crystal structures of n = 1 layered perovskites ranked from left to right by highest out-of-plane conductivity to lowest [88]. **c** Time-resolved photoluminescence (TRPL) spectra for the FuMA- and THFMA-based films [39]. **d** The photos of (PEA)_2_MA_4_Pb_5_I_16_, (3AMP)_2_MA_4_Pb_5_I_16_ and (CA)_2_MA_4_Pb_5_I_16_ perovskite films immersed in water at 1, 60, 120, and 300 s; Immersion time of CA-based 2D perovskite films with different *n*-values in water before they decompose [89]

In addition to the thiophene and benzene derivatives, a few other fused-ring-based aromatic derivatives were also used as bulk organic spacer in 2D RP perovskite. In 2019, Wang et al*.* used 4-(aminoethyl)-pyridine (4AEPY) as organic spacer to fabricate RP-type 2D PSCs (n = 5) [86]. They found that the additional non-covalent interaction between nitrogen atom on the pyridyl unit and Pb^2+^ could retard the crystallization rate of 2D perovskite, resulting in high-quality 2D RP perovskite films and devices. Liu et al*.* reported two multiple-ring-based organic aromatic amines, namely 1-naphthalenemethylammonium (NpMA) and 9-anthracenemethylammonium (AnMA), as the spacers to fabricated 2D PSCs and achieved a high *V*_*OC*_ of 1.24 V with a champion PCE of 17.25% for NpMA-based device [87]. Compared to the AnMA-based perovskite, the 2D perovskite of (NpMA)_2_(MA)_n−1_Pb_n_I_3n+1_ (n = 4) exhibited ultrafast exciton migration (within 7 ps) between 2D phases and 3D-like phases confirmed by TA spectra (Fig. 7a), which could help explain its outstanding photovoltaic performance. Beyond that, naphthalene, pyrene, and perylene derivatives as the organic spacers were subsequently used to fabricate the 2D perovskite (n = 1) [88]. The general chemical formula of this series of cations was defined as “Aromatic-O-Linker-NH_3_” where the aromatic moiety was naphthalene, pyrene, or perylene and the linker was ethyl, propyl, or butyl. Comparing their corresponding 2D perovskite structures of (aromatic-O-linker-NH_3_)_2_PbI_4_, the authors found that the out-of-plane conductivity gradually decreases with the increased inorganic layer spacing. In addition, the stacking model of the organic spacers also plays a crucial role for out-of-plane conductivity in the 2D perovskite. The “edge-to-face” stacking model of the pyrene molecules is more favorable than the “edge-to-edge” stacking type for improving the out-of-plane conductivity (Fig. 7b). However, the (perylene-O-ethyl-NH_3_)_2_PbI_4_ stacking as “edge-to-edge” model exhibited the highest out-of-plane conductivity, mainly because of its better energy level arrangement. The improved out-of-plane conductivity resulted in an efficiency of 1.38% for the corresponding 2D PSCs (n = 1).

Furan derivative, Furan-2-yl methanaminium iodide (FuMAI), was also developed for constructing 2D perovskite solar cells by Zheng and coworkers [90]. The (FuMA)_2_(MA)_4_Pb_5_I_16_ perovskite films fabricated in ambient air via an additive-assisted film forming technique afforded a high PCE of 15.24%, which is comparable with that prepared in an inert atmosphere (PCE = 15.66%). Liu et al*.* reported two structurally similar organic spacers with conjugated and unconjugated unites, namely FuMACl and (tetrahydrofuran-2-yl) methanaminium chloride (THFMACl), respectively, for 2D perovskites (n = 4) [39]. Compared with the perovskite film based on unconjugated spacer THFMA (*τ*_avg_ = 0.42 μs), the 2D RP perovskite film fabricated using conjugated FuMA spacer exhibited longer average carrier lifetime of 18.03 μs from TRPL spectra (Fig. 7c). The corresponding FuMA-based 2D perovskite device shows an enhanced efficiency of 18.00% in comparison with 13.79% of THFMA-based device. The ultralong carrier lifetime and enhanced PCE could be attributed to the enlarged dielectric constant, decreased *E*_b_, and reduced electron − phonon coupling coefficients of FuMA-based 2D perovskite film. Moreover, the FuMA-based 2D perovskite film showed better moisture and light stability than that of 3D MAPbI_3_ film.

Recently, Yuan et al*.* reported a carbazole derivative, (9H-carbazol-9-yl)butyl-1-ammonium (CA) cation, as the organic spacer for 2D perovskite [89]. Compared with the PEA- and 3AMP-based 2D perovskite films (n = 5), the CA-based 2D perovskite film (n = 5) exhibited ultrahigh moisture stability and did not decompose after soaking in water for 300 s, which could be attributed to the hydrophobic nature of π-conjugated CA cations and the unique gradient phase distribution with the hydrophobic low-(n)-value phase concentrated on the surface of perovskite film (Fig. 7d). In addition, the vertical crystal orientation was also obtained by introducing the NH_4_SCN additive, resulting in enhanced charge transport and suppressed non-radiative recombination of the 2D perovskite film. As a result, the CA-based 2D PSCs achieved a notable PCE of 18.23% and can retain more than 85% of the initial PCE after aging 2000 h under 65% RH at 25 °C.

### Aromatic Spacers for DJ 2D PSCs

Recently, DJ type perovskites containing divalent organic spacers have been drawing widespread attention as promising materials for solar cell applications. Compared with RP type perovskite, 2D DJ perovskite has several main advantages that make it more suitable to achieve the ideal device performance in PSCs [2, 6, 19, 58, 63, 64], for example, (1) *Well-aligned octahedral arrangement*. The monovalent organic spacers in RP perovskites lead to the half-octahedral displacement between the adjacent inorganic slabs. However, no such displacement exists in 2D DJ perovskites due to the vertical bonds with inorganic layers. (2) *Shortened interlayer distance*. The interlayer distance between the inorganic layers directly influences the charge transport capability and device performance. The DJ type perovskite has a shorter interlayer distance than the corresponding RP type perovskite providing enhanced charge transport capability between two inorganic layers. (3) *Eliminated van der Waals gap*. Generally, the tails of monovalent cations interact by van der Waals interactions to form the van der Waals gaps. But for DJ perovskite, there is no such interactions (Fig. 2c). The elimination of the van der Waals gap can improve the structural stability of 2D DJ perovskite. Benefitting from the advantages described above, we believe that 2D DJ PSCs perovskites should have better photovoltaic performance than 2D RP perovskites-based devices. Although 2D DJ perovskite has shown great potential in solar cells with improved stability, the development of new organic spacers is highly necessary to further boost their photovoltaic performance. However, the studies related to 2D DJ PSCs are still relatively lacking, probably because the decreased solubility of the divalent spacers.

**Fig. 8:**
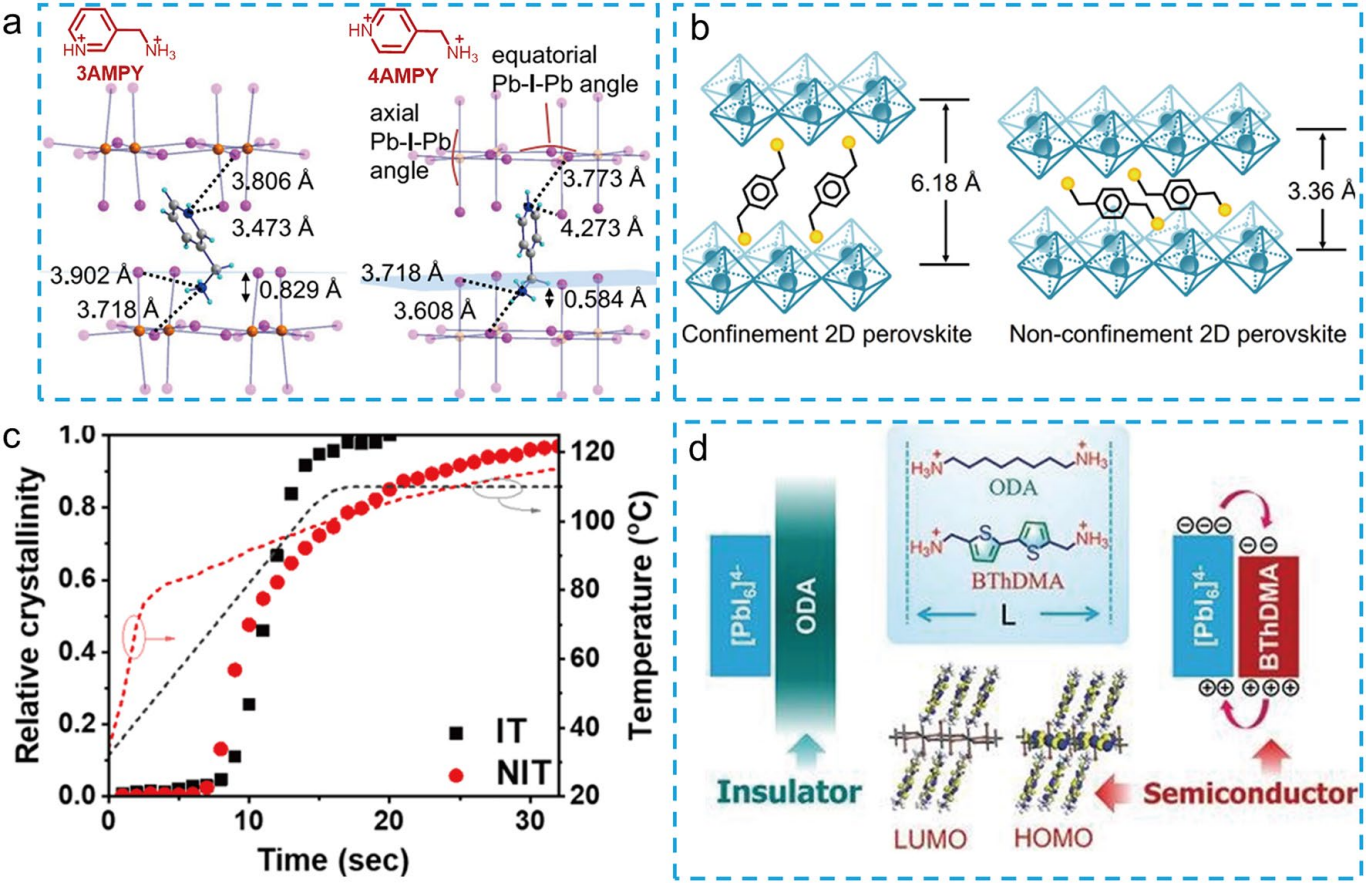
**a** Structural comparison of (3AMPY)(MA)Pb_2_I_7_ and (4AMPY)(MA)Pb_2_I_7_ [38]. **b** Schematic crystal structures of confinement and non-confinement 2D perovskite [42]. **c** Plot of relative crystallinity (symbol) and temperature traces (dashed line) vs time [94]. **d** Chemical structures of ODA and BThDMA; DFT-calculated HOMO and LUMO of the optimized 2D perovskite structures of (ODA)PbI_4_ and (BThDMA)PbI_4_ [44]

Inspired by the successful application of 3-(aminomethyl) piperidine (3AMP) and 4-(aminomethyl)piperidine (4AMP) in 2D DJ PSCs, Kanatzidis et al*.* reported two novel aromatic cations with conjugated units, namely 3-(aminomethyl)pyridine (3AMPY) and 4-(aminomethyl)pyridine (4AMPY), to explore their effects on the crystal structure and photovoltaic properties of 2D DJ perovskite[38]. The conjugated AMPY spacers with higher dielectric constant and rigidity structure guarantee excellent charge transport and outstanding structure stability of 2D perovskite films due to its shorter distance between adjacent inorganic layers in comparison with the unconjugated AMP based-counterparts. Moreover, the symmetry of spacers also has significant effects on the perovskite crystal structures. The relatively poor symmetry of the 3AMPY results in a slight shift of the two adjacent inorganic layers, while no such shift in the 2D perovskite crystal structure with more symmetrical 4AMPY spacer (Fig. 8a). This structural difference could impact the optical properties of 2D perovskite film, such as the shifting of absorption band and the changing of band gap. As a result, The PSCs based on (3AMPY)(MA)_3_Pb_4_I_13_ obtained an efficiency 9.2%, which is larger than that of the 4AMPY-based device (PCE = 5.69%). In addition, England et al*.* used pyridinium ethylamine iodide (PyrEAI) and imidazolium ethylamine iodide (ImEAI) as organic spacers for 2D perovskites (n = 1), obtaining the highest PCE (1.83%) for pure n = 1 2D PSCs at that time [91]. Compared to PEA, PyrEA and ImEA had a lower charge density, resulting in smaller twist angle of [PbI_6_]^4−^, which reduced the band gap of the 2D perovskites and increased the *Jsc* of the devices. In 2019, Grätzel et al*.* first reported a 1,4-phenylenedimethanammonium (PhDMA) spacer for DJ type PSCs ((PhDMA)FA_2_Pb_3_I_10_), achieving an excellent device with PCEs over 7% and improved long-term stability in humid ambient air condition [40]. Meanwhile, Etgar et al*.* found that PhDMA-based 2D DJ perovskite (n = 10) with mixed A-site cations exhibited high crystal orientation without additives or pretreatment [41]. After careful device optimization, the 2D DJ PSCs showed a high PCE of 15.6%. After that, Liang et al*.* also systematically studied the structural and optoelectronic properties of 2D DJ perovskites (PhDMA)(MA)_n−1_Pb_n_I_3n+1_ [42]. They revealed that nonconfinement structure could be obtained in large-n value perovskite films (n > 2), which showed a shorter distance of 3.4 Å among 2D QWs and gave rise to significantly diminishing quantum confinement (Fig. 8b). Consequently, an impressive efficiency of over 11% was achieved, along with enhanced ambient stability. More recently, Liu et al*.* innovatively created an improved preparation technique for the (PhDMA)(MA)_n−1_Pb_n_I_3n+1_ (n = 4) perovskite film and achieved a high efficiency 2D PSCs with PCE of 15.81% [92]. The high-quality perovskite film with enhanced charge transport and perpendicular orientation was obtained by quickly removing the solvent via the hot-casting process. The fluorinated PhDMA spacer, namely 4F-PhDMA, developed by Liu et al. shows multiple noncovalent interactions in the corresponding 2D perovskites single crystal, including CH···F and F···I interactions, which enhance the structural stability [43]. The multiple noncovalent interactions can reduce the dissociation energy of 4F-PhDMA-based 2D perovskite, which increases the efficiency of corresponding 2D PSCs to 16.62%. Importantly, the introduced fluorine in aromatic moiety can enhance the hydrophobicity of the perovskite film, leading to significantly improved moisture stability. As an analogue of phenyl group, naphthalene has also been used to construct bulky conjugated spacer. In 2022, Satapathi et al*.* reported a novel aromatic spacer, namely 1,5-diaminonaphthalene (NDA), for 2D DJ PSCs [93]. The (NDA)(MA)_3_Pb_4_I_13_ 2D perovskite film treated with NH_4_SCN exhibited excellent crystallinity, substantially reduced trap density and enhanced charge carrier mobility. Eventually, a high efficiency of 15.08% was achieved with excellent moisture stability.

Thiophene-based diammonium cations are another famous spacer for fabricating 2D DJ perovskites. Liu et al*.* reported a new thiophene derivative, 2,5-thiophenedimethylammonium iodide (ThDMAI), for 2D DJ PSCs (n = 5) [95]. The authors systematically investigated the effect of precursor solvents (DMF/DMSO) on film quality. Because of the strong coordination between DMSO and PbI_2_, they found that the tiny amount of DMSO could adjust the crystallization rate of perovskite film. The obtained film showed a preferred vertical orientation, enlarged grain size, and more efficient charge transport. As a result, the optimized PSCs exhibited a champion PCE of 15.75% with dramatically improved environmental stability. Later, a fused-thiophene-based spacer, namely thieno[3,2-b]thiophene-2,5-diyldimethanaminium (TTDMA), was further developed by Liu et al*.* for the application of 2D DJ PSCs [96]. The 2D DJ perovskite film using TTDMA as the spacer displayed an enlarged crystal size with preferred vertical orientation owing to the extended π-conjugation length of TTDMA and the template effect enforced by strong intermolecular interaction. Compared with ThDMA-based PSCs (PCE = 15.3%), the (TTDMA)MA_n−1_Pb_n_I_3n+1_ (n = 4) PSCs showed higher efficiency of 18.82% after careful device optimization. In 2022, Chou et al*.* proposed a new non-isothermal (NIT) crystallization method to improve the film quality of 2D DJ perovskite [94]. The perovskite films prepared using the NIT method exhibited better morphology with dense and large grain size and a more effective network-like charge transport channel, resulting in a PCE of 17.86% with a fill factor (FF) of up to 84.32% for PhDMA-based 2D DJ PSCs and a higher PCE of 19.87% with much higher FF of 86.16% in TTDMA-based systems. In addition, a series in-depth mechanism studies were performed, and the results showed that the slow secondary crystallization induced by the NIT process was the main reason for the significantly improved film quality (Fig. 8c).

Typically, one of main role of the organic spacers in 2D perovskites is to balance the charge state. The interactions between the organic spacer and inorganic are largely unexplored. Recently, Liu et al*.* reported an organic semiconductor spacer, namely [2,2'-bithiophene]-5,5'-diyldimethanaminium (BThDMA), for 2D DJ PSCs [44]. Compared with the non-conjugated spacer of aliphatic octane-1,8-diaminium (ODA) with similar length, the multiple conjugated BThDMA organic spacer layers showed intensive orbital interactions with adjacent inorganic layers (Fig. 8d), resulting in enlarged carrier mobility and electrical conductivity. As a result, the device based on (BThDMA)MA_n−1_Pb_n_I_3n+1_ (n = 5) exhibited a high PCE of 18.1% with negligible hysteresis, which is much higher than that of the ODA-based device (PCE = 12.3%). The orbital interaction of organic spacer and inorganic layer reported in this work provide a novel and important insight on understanding the role of organic semiconductor spacers in 2D perovskites. Ge et al. reported a donor–acceptor–donor type organic spacer, namely DPP-2 T, for 2D DJ perovskite and achieving an impressive PCE of 18.6% for corresponding PSCs [97]. Notably, the perovskite film with this novel spacer shows a flattened energy landscape of the QWs, which brings about weaker quantum confinement and enlarged carrier diffusion lengths.

## Conclusion and Outlook

In summary, we have reviewed the recent development of 2D PSCs based on different aromatic spacers. 2D perovskites have become emerging photovoltaic materials due to their unique photovoltaic and photophysical properties. However, the PCEs of these 2D PSCs still lag behind those of state-of-the-art 3D PSCs, primarily due to strong quantum and dielectric confinement effects in 2D perovskites. One possible solution to address this limitation and improve device performance is to employ aromatic spacers, which could increase the dielectric constant of the corresponding 2D perovskites and reduce their exciton binding energy. The aromatic spacers could play a crucial role in adjusting film quality, crystal orientation, charge transport capability, exciton binding energy, and overall device performance. Thus, the rational design and selection of organic spacers can effectively improve the efficiency of 2D PSCs.

Based on the contents of this review, we propose a few design strategies of organic spacers in 2D PSCs. Firstly, aromatic spacers with high dielectric constant can reduce the dielectric mismatch between organic and inorganic layers in 2D perovskite films, resulting in reduced exciton binding energy and improved charge transport capability. Aromatic spacers with large dipole moment typically exhibit high dielectric constant. Secondly, introducing appropriate intermolecular interactions can significantly affect the perovskite crystal structure and optoelectronic properties. The stacking model of organic spacers in 2D perovskite should be carefully considered. Thirdly, long conjugated semiconductor spacers with strong orbital interactions with the adjacent inorganic layer in 2D perovskites could improve device performance by reducing or eliminating quantum and dielectric confinement, thus maintaining their superior stability. Fourthly, diammonium aromatic spacers for DJ perovskites can eliminate the van der Waals gap and shorten the distance between two adjacent inorganic layers, creating more opportunities to achieve excellent photovoltaic properties and high structure stability in theory than RP type perovskites.

These design strategies are promising but also challenging to implement due to the solubility issue of diammonium aromatic spacers and the complex synthesis steps and poor solubility of organic semiconductor spacers with elongated conjugated structures. Balancing the spacer's semiconductor properties with its solubility presents a challenge for researchers. Moreover, modulating the intermolecular interaction of the spacers in 2D perovskite can effectively improve their crystal quality and device performance, but it can also induce undesirable aggregation of the aromatic spacers in 2D perovskite, further interfering with their interaction with inorganic layers. Therefore, precisely modulating the interaction between aromatic spacers in 2D perovskite requires the assistance of single-crystal technology, which is challenging but imperative. Researchers should devote more attention to the single-crystal synthesis of 2D perovskites and analyze their relationship with perovskite film properties.

In addition, the film quality of 2D perovskites also plays a crucial role in their photovoltaic performance. Therefore, developing effective film preparation techniques, such as the hot-casting method, non-isothermal (NIT) crystallization protocol, vapor processing, etc., to regulate the crystallization kinetics and improve the film quality is highly necessary.
